# Real-space observation of polarization induced charges at nanoscale ferroelectric interfaces

**DOI:** 10.1126/sciadv.adu8021

**Published:** 2025-06-13

**Authors:** Masaya Takamoto, Satoko Toyama, Takehito Seki, Toshihiro Futazuka, Scott D. Findlay, Yuichi Ikuhara, Naoya Shibata

**Affiliations:** ^1^ Institute of Engineering Innovation, School of Engineering, The University of Tokyo, Tokyo 113-8656, Japan.; ^2^ Murata Manufacturing Co. Ltd., Shiga 520-2393, Japan.; ^3^ PRESTO, Japan Science and Technology Agency, Saitama 332-0012, Japan.; ^4^ School of Physics and Astronomy, Monash University, Melbourne, VIC 3800, Australia.; ^5^ Nanostructures Research Laboratory, Japan Fine Ceramic Center, Nagoya 456-8587, Japan.; ^6^ Quantum-Phase Electronics Center (QPEC), The University of Tokyo, Tokyo 113-8656, Japan.

## Abstract

Unique electrical properties emerging at nanoscale ferroelectric interfaces originate from the polarization induced charges. However, real-space characterization of polarization induced charges at nanoscale ferroelectric interfaces has been extremely challenging. Here, directly observing the nanoscale electric field by tilt-scan averaged differential phase contrast scanning transmission electron microscopy enables us to measure the spatially varying total charge density profiles across both head-to-head and tail-to-tail domain walls in a ferroelectric crystal. Combined with atomic column displacement measurements, the spatial distribution of polarization bound charges and screening charges across the domain walls can be disentangled. Our results reveal the true charge states of the nanoscale ferroelectric interfaces, providing an opportunity for experimentally exploring the interplay between atomic-scale local polarization structures and their charge states in ferroelectric interfaces.

## Teaser

Local charges at nanoscale ferroelectric interfaces are observed in real space by transmission electron microscopy.

## INTRODUCTION

The structures and properties of domains and domain walls are of great importance in determining the properties of ferroelectric materials (*1*, *2*). Recent advances in (scanning) transmission electron microscopy [(S)TEM] have enabled direct characterization of domains and domain walls at atomic dimensions. Seminal works by Jia *et al.* (*3*) demonstrated that high-resolution TEM can directly map local polarization changes across heterointerfaces, domain walls (*4*), and flux-closure structures (*5*) at the single–unit cell level. STEM has also been intensively used to directly map local polarization changes across domain walls (*6*), heterointerfaces (*7*), and flux-closure structures (*8*, *9*) and recently found topological polar structures (*10*, *11*). All these local polarization analyses rely on atomic column displacement measurements with precision on the order of picometers. On the basis of the experimental atomic column displacements, local polarization is estimated unit cell by unit cell assuming isolated ionic charge or Born effective charge of each atomic column from density functional theory calculations.

In the theory of ferroelectrics, if two adjacent domains A and B with polarizations of P_A_ and P_B_ are separated by an atomically sharp domain wall, then the polarization bound sheet charge density, σ_p_, should form at the domain wall according to the following formula (*12*)

$$\sigma_{p}=\left(P_{B}-P_{A}\right)\cdot n_{A}$$ (1)

where **n**_A_ is the wall normal unit vector pointing inside domain A. If the domain wall has finite width, then it is better described via a polarization bound volume charge density, ρ_p_, in the vicinity of the domain wall according to the following formula

$$\rho_{p}=-\nabla \cdot P$$ (2)

where ∇· is the divergence operator. It is anticipated that the electrostatic energy of the polarization bound charge distribution is minimized via screening by free carriers or charged point defects. Screening charges have been postulated to be a source of unique electrical properties of domain walls (*12*). Because such domain walls are mobile and, thus, their position and alignment can be controlled, they can be used for nanoscale devices (*13*). Characterizing the actual charge state of domain walls, including screening charges, is thus of critical importance for understanding the origin of domain wall properties. However, the abovementioned atomic column displacement measurements using atomic-resolution (S)TEM have not been able to directly characterize the actual charge state of domain walls.

Other electron microscopy techniques, such as electron holography, differential phase contrast (DPC) STEM or four-dimensional STEM, have the potential to characterize electric fields and the associated charge distributions at very high spatial resolution (*14*, *15*). However, there have been several difficulties for directly observing local charge states across domain walls in ferroelectrics. One major problem is the diffraction contrast due to the breaking of Friedel’s law caused by dynamical diffraction in non-centrosymmetric crystals (*16*). This effect causes strong additional contrast in the experimental images, hindering our interpretation of the domain walls’ true electric field and charge distribution information.

In recent years, the tilt-scan averaged DPC (tDPC) STEM technique has been developed (*17*) and has proven to be very effective for minimizing diffraction contrast in DPC STEM images (*18*). In this technique, the incident-beam-tilt conditions are systematically changed while the incident electron probe is stationary at the same sample position. The diffraction patterns under multiple beam-tilt conditions are then averaged on the detector plane before evaluating the DPC STEM signal. The tDPC image is thus effectively the average of many DPC images with different incident beam directions. This technique has enabled real-space visualization of nanoscale electromagnetic fields and accumulated charges at crystalline interfaces by suppressing strong diffraction contrast (*19*, *20*). Moreover, the imaging conditions for reducing the diffraction contrast due to the breaking of Friedel’s law have been recently explored (*21*), where it was shown that the crystallographic imaging direction and the sample thickness are key factors for reducing the diffraction contrast in DPC STEM. In the present study, by combining the recently developed technique and well-controlled experimental conditions, we observe polarization-induced charges across domain walls in a model ferroelectric crystal (LiTaO_3_) in real space.

## RESULTS

### Atomic-resolution STEM of head-to-head and tail-to-tail domain walls in LiTaO_3_

**Fig. 1.**
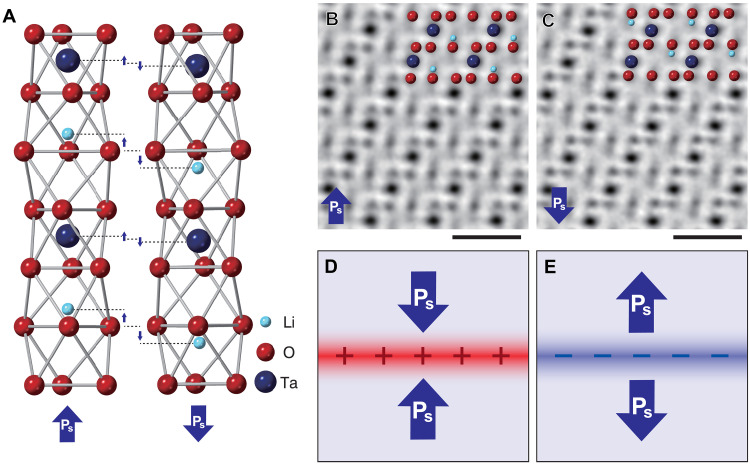
Crystal structure and atomic resolution OBF STEM images of ferroelectric LiTaO_3_ crystal. (**A**) Crystal structure model of LiTaO_3_. Due to the relative shift of cations and anions along the <0001> crystallographic axis as indicated by the small arrows, the spontaneous polarization (P_s_) points either along the [0001] or [000$\bar{1}$ ] directions as indicated by the large arrows. (**B** and **C**) Atomic-resolution OBF STEM images of LiTaO_3_ observed from the [11$\bar{2}$ 0] direction. Ta, Li, and O atomic columns appear as dark contrast. By using the relative positions of Ta atomic columns with respect to the O atomic layers along the <0001> axis, the direction and magnitude of spontaneous polarization can be estimated from the OBF images. Scale bars, 0.5 nm. (**D** and **E**) Schematic illustrations of head-to-head (H-H) and tail-to-tail (T-T) domain walls, respectively. These domain walls carry opposite polarization bound charges at their cores.

In this study, we observe ferroelectric LiTaO_3_ as a model sample. LiTaO_3_ is a uniaxial trigonal ferroelectric which is widely used in nonlinear optics and electro-optics (*22*). Because Ta and Li ions are shifted relative to O ions along the <0001> axis, as schematically shown in Fig. 1A, the spontaneous polarization points either along the [0001] or [000$\bar{1}$ ] axes. Figure 1 (B and C) shows atomic resolution optimum bright-field (OBF) STEM images (*23*) of LiTaO_3_ observed along the [11$\bar{2}$ 0] direction. OBF STEM reconstructs a high contrast image by applying frequency filters to maximize the signal-to-noise ratio on simultaneously obtained segmented detector STEM images and, therefore, enables atomic-structure imaging of materials under very low–electron dose conditions (*24*). In the OBF image, all the atomic columns (Ta, Li, and O) are visualized as dark contrast, and we can directly determine the direction and amount of spontaneous polarization from the relative positions of the Ta and O atomic columns. As schematically shown in Fig. 1 (D and E), two types of 180° domain walls can exist in LiTaO_3_: head-to-head (H-H) and tail-to-tail (T-T) domain walls. These domain walls are known as charged domain walls (*12*) because, according to formula 1, they carry opposite polarization bound charges owing to the change of the normal component of spontaneous polarization across the walls. Figure 1 (D and E) depicts the H-H and T-T domain walls as normal to the polarization direction, but, in practice, the domain wall normal tends to be inclined from the polarization direction, which reduces the electrostatic energy.

**Fig. 2.**
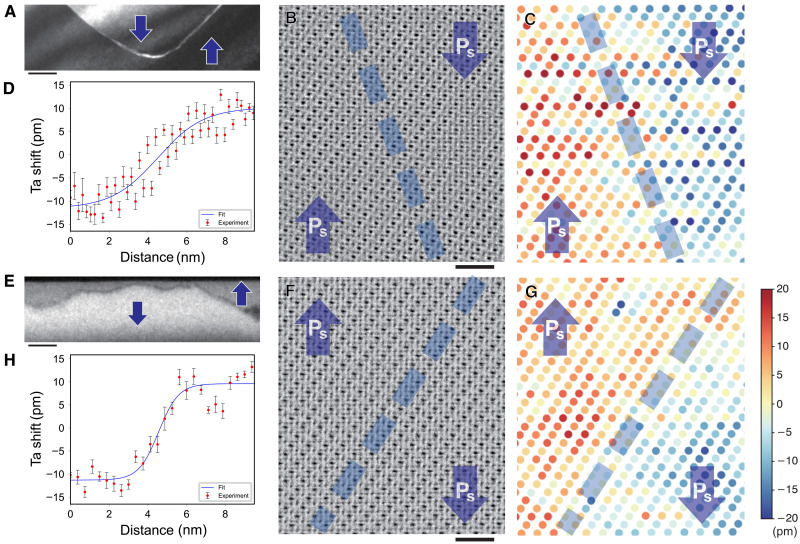
Atomic resolution OBF STEM images of H-H and T-T domain walls in ferroelectric LiTaO_3_ crystal. (**A**) A typical DF TEM image of an H-H domain wall. Scale bar, 50 nm. (**B** and **C**) Atomic resolution OBF STEM image of an H-H domain wall and corresponding polarization map, respectively. Scale bars, 1 nm. The relative shifts of Ta atomic columns with respect to the adjacent O atomic layers are precisely measured to form the polarization map. The domain wall position is indicated by the dashed line. (**D**) Line profile of the polarization map across the H-H domain wall. The domain wall width is estimated to be 8.2 nm. (**E**) A typical DF TEM image of a T-T domain wall. Scale bar, 50 nm. (**F** and **G**) Atomic resolution OBF STEM image of a T-T domain wall and corresponding polarization map, respectively. The domain wall position is indicated by the dashed line. (**H**) Line profile of the polarization map across the T-T domain wall. The domain wall width is estimated to be 3.8 nm.

Figure 2A shows a typical low-magnification dark-field (DF) TEM image of an H-H domain wall in LiTaO_3_. The domain wall separating upper and lower domains is clearly observed. Atomic-resolution OBF STEM image and corresponding atomic-scale polarization map of an H-H domain wall are shown in Fig. 2 (B and C), respectively. To construct the atomic-scale polarization map, relative Ta atomic column positions along the <0001> axis with respect to the adjacent O layers were precisely measured. It is seen that the polarization is reversed across the domain wall, confirming the H-H configuration. It is noted that the domain wall is not atomically sharp but has finite width. From the line profile of the polarization map across the domain wall shown in Fig. 2D, the domain wall width is estimated to be about 8.2 nm. The details on the width estimation are given in the Supplementary Materials. It has also been shown by both theory and experiments that the domain wall widths of the 180° domain walls are dependent on the wall plane orientation and that the width of the H-H and T-T domain walls is much wider than that of walls lying parallel to the polarization vectors, reflecting the charge state of the domain walls (*4*, *25*).

Figure 2E shows a typical low-magnification DF TEM image of a T-T domain wall. Atomic-resolution OBF STEM image and corresponding atomic-scale polarization map of a T-T domain wall are shown in Fig. 2 (F and G), respectively. It is seen that the polarization is reversed across the domain wall, confirming the T-T configuration. The domain wall width is estimated to be about 3.8 nm from the line profile of the polarization map shown in Fig. 2H. This T-T domain wall has a narrower width than the H-H domain wall. We have checked domain wall widths from other regions in the field of view and found consistently thinner wall width in T-T domain walls compared with that in H-H domain walls.

### tDPC STEM of H-H and T-T domain walls in LiTaO_3_

It has been reported that DPC STEM observation from the <11$\bar{2}$0 > axis and making the specimen thickness less than 70 nm can effectively reduce diffraction contrast that originates from the breaking of Friedel’s law in LiTaO_3_ (*21*). In this study, we observe H-H and T-T domain walls by tDPC STEM from the <11$\bar{2}$0 > axis at a specimen thickness of less than 70 nm. The sample preparation procedures and optical conditions for tDPC STEM are summarized in Materials and Methods in the Supplementary Materials. Here, based on the convergent beam electron diffraction (CBED) patterns from upper and lower domains, as shown in figs. S1 and S2, the domain wall types (H-H or T-T) are unambiguously determined.

**Fig. 3.**
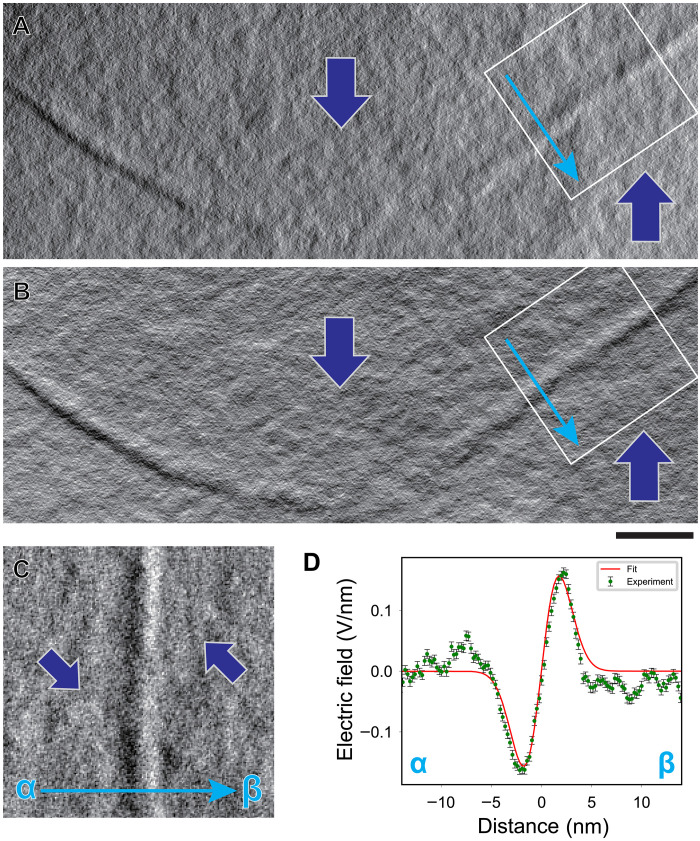
tDPC STEM images of an H-H domain wall in ferroelectric LiTaO_3_ crystal. (**A** and **B**) *X*- and *Y*-component electric field images, respectively, of an H-H domain wall obtained by tDPC STEM. Scale bar, 20 nm. Using the *X*- and *Y*-component images in (A) and (B), the horizontal electric field component image perpendicular to the domain wall plane is constructed as shown in (**C**). The bright and dark image contrast corresponds to the horizontal electric field component pointing rightward or leftward, respectively. (**D**) Projected line profile of (C) across the H-H domain wall from α to β. The red line shows the fitted profile using the first derivative of a Gaussian function.

Figure 3 (A and B) shows horizontal (*X*) and vertical (*Y*) electric field component images of an H-H domain wall observed by tDPC STEM. Line-like contrast corresponding to the H-H domain wall is seen in both images. Except for the line-like contrast at the domain wall and the fine speckle-like contrast due to the surface roughness of the TEM specimen, the contrast in both images is uniform in the upper and lower domains, indicating that diffraction contrast has been effectively eliminated by the tDPC technique. Figure 3C, a magnified image of the electric field component perpendicular to the H-H domain wall constructed from the field components in the white rectangular region in Fig. 3 (A and B), shows that the image contrast reverses across the domain wall, from dark on the left to bright on the right. This contrast reversal is clearly seen in the corresponding line profile shown in (D). The electric field strength increases in the vicinity of the domain wall but reverses direction at the center of the domain wall. This profile indicates that the electric field is emanating from the domain wall center into each domain interior, evidencing the presence of positive bound charges at the core of the H-H domain wall.

**Fig. 4.**
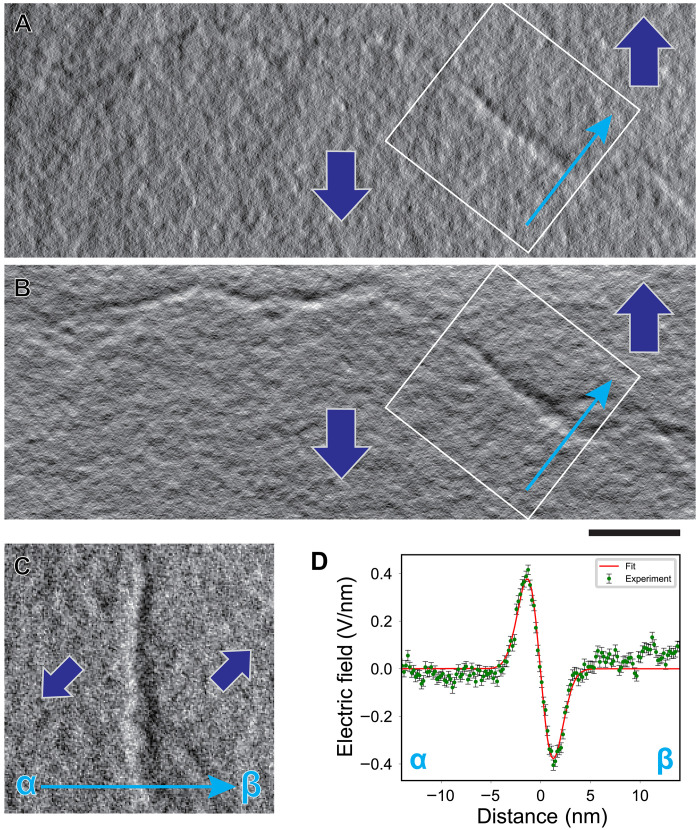
tDPC STEM image of a T-T domain wall in ferroelectric LiTaO_3_ crystal. (**A** and **B**) *X*- and *Y*-component electric field images, respectively, of a T-T domain wall obtained by tDPC STEM. Scale bar, 20 nm. Using *X*- and *Y*-component images in (A) and (B), the horizontal electric field component image perpendicular to the domain wall plane is constructed as shown in (**C**). The bright and dark image contrast corresponds to the horizontal electric field component pointing rightward or leftward, respectively. (**D**) Projected line profile of (C) across the T-T domain wall from α to β. The red line shows the fitted profile using the first derivative of a Gaussian function.

Figure 4 (A and B) shows *X* and *Y* electric field component images of a T-T domain wall observed by tDPC STEM. Line-like contrast corresponding to the T-T domain wall is seen in both images. Figure 4C, a magnified image of the electric field component perpendicular to the T-T domain wall constructed from the field components in the white rectangular region in Fig. 4 (A and B), again shows that the image contrast reverses across the domain wall. However, now the contrast reverses from bright on the left to dark on the right, the opposite of what was seen for the H-H domain wall. This contrast reversal is also clearly seen in the corresponding line profile shown in (D). This profile indicates that the electric field is converging from domain interiors into the domain wall center, evidencing the presence of negative bound charges at the core of the T-T domain wall.

The tDPC imaging suppresses diffraction contrast, minimizing that source of potentially confounding contrast (*26*). However, the effect of (projected) mean-inner-potential changes, which can originate from a decrease in local sample thickness due to preferential sample milling and/or from a decrease in local atomic density due to low atomic density interface core structures, can still be superimposed on the tDPC images from crystalline interfaces (*20*). As shown in fig. S3, we find no appreciable reduction in thickness or local atomic density across the domain wall by electron-energy-loss spectroscopy and annular dark-field (ADF) STEM imaging. Further, such changes would produce similar DPC contrast in both H-H and T-T boundaries, whereas our images show reversed contrast between H-H– and T-T–type domain walls. Thus, we consider the effect of any (projected) mean-inner-potential changes present to be minimal in these tDPC observations.

To convert the experimental electric field profiles into charge density profiles through the differential form of Gauss’s law, we need to minimize the effect of noise in the experimental profiles. Here, we simply use the first derivative of a Gaussian as a convenient functional form with which to fit the experimental electric field profiles and construct the charge density profile across the domain wall based on the fit. Details for the fitting procedures are given in the Supplementary Materials. The fitted electric field profiles are shown as red lines in Figs. 3D and 4D, where we see that the assumed functional form is a good approximation to the data.

**Fig. 5.**
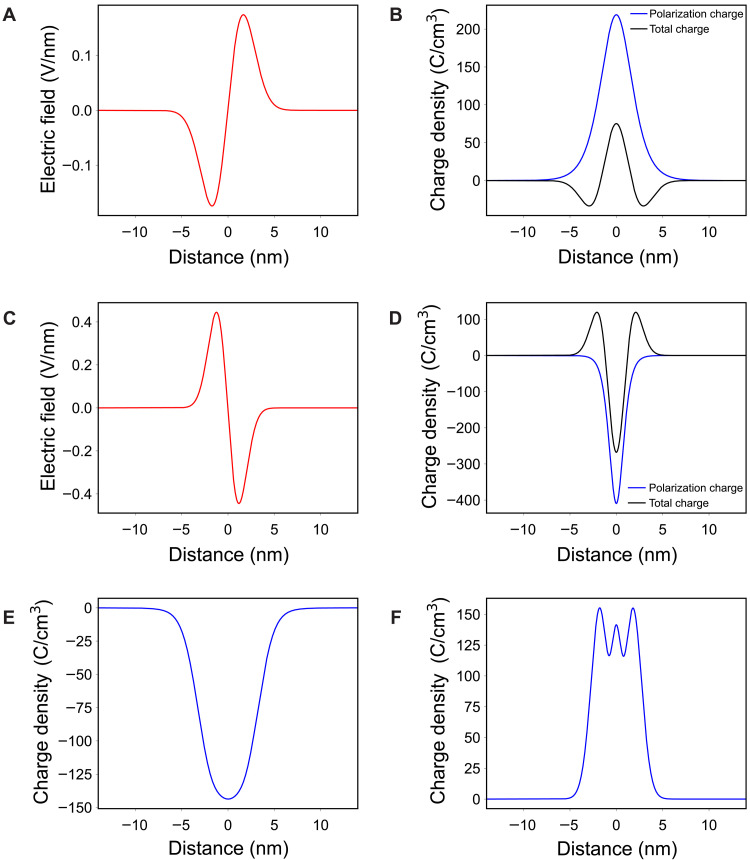
Fitted electric field and charge density profiles of the H-H and T-T domain walls. (**A** and **B**) Fitted electric field and corresponding charge density profiles of the H-H domain wall (with finite probe size effects deconvolved). As seen in (B), the core of the domain wall is positively charged, surrounded on both sides by negatively charged layers. (**C** and **D**) Fitted electric field and corresponding charge density profiles of the T-T domain wall (with finite probe size effects deconvolved). As seen in (D), the core of the domain wall is negatively charged, surrounded on both sides by positively charged layers. Using the OBF STEM estimated domain wall width and spontaneous polarization of LiTaO_3_ at room temperature (*29*), polarization bound charge profiles are estimated and plotted as shown by the dashed lines in (B) and (D). By subtracting the bound charge profiles from the total charge profiles, screening charge profiles of the H-H and T-T domain walls are plotted as shown in (**E**) and (**F**). The small peaks in the vicinity of the domain wall core in (F) may be an artefact caused by the errors in our profile fitting and sample thickness estimation.

Figure 5 (A and B) shows the fitted electric field profile and corresponding total charge density profile (black solid line) of the H-H domain wall, respectively. In the present tDPC experiments, the finite probe size was about 1 nm. The results in Fig. 5 (A and B) have removed the probe blurring effect, including that resulting from beam-tilting, by deconvolving the probe size from the fitted electric field and charge density profiles. The deconvolution procedures are given in the Supplementary Materials. As shown in fig. S4, deconvolution makes the profiles slightly sharper and the intensity peaks slightly higher. The total charge density profile in Fig. 5B (black solid line) shows a strong peak of positive charge at the center of the H-H domain wall, which corresponds to the positive polarization bound charge. From the positive bound charge width in the profile, the domain wall width is estimated to be about 3.1 nm. It is clearly seen that there are negative screening charge layers surrounding both sides of the positive bound charge. These negative charges should accumulate to reduce the excess electrostatic energy of the positive bound charge. Figure 5 (C and D) shows the fitted electric field profile and corresponding total charge density profile (black solid line) of the T-T domain wall, respectively, again after correcting from the probe blurring effect. There is a strong peak of negative charge at the center of the T-T domain wall, which corresponds to the negative polarization bound charge. From the negative bound charge width in the profile, the domain wall width is estimated to be about 2.2 nm. It is seen that there are positive screening charge layers surrounding both sides of the negative bound charge.

Previous reports (*27*, *28*) showed that the major negatively charged point defects in near-stoichiometric LiTaO_3_ single crystal are Li^+^ vacancies ( $V_{\text{Li}}^{-}$ ), which have formal charge of −1. Therefore, the main compensating negative charge may be $V_{\text{Li}}^{-}$ for the H-H domain wall. However, it is very hard to directly detect $V_{\text{Li}}^{-}$ by STEM imaging techniques. On the other hand, the major positively charged point defects are reported to be antisite Ta^5+^ in Li^+^ sites ( ${\text{Ta}}_{\text{Li}}^{4+}$ ), which has formal charge of +4. Therefore, the main compensating positive charge may be ${\text{Ta}}_{\text{Li}}^{4+}$ for the T-T domain wall. To confirm this, an ADF STEM image was obtained at the T-T domain wall as shown in fig. S5. The ADF STEM image shows strong image contrast at some Li sites, consistent with the accumulation of ${\text{Ta}}_{\text{Li}}^{4+}$ in the vicinity of the T-T domain wall.

## DISCUSSION

By the atomic displacement analysis using atomic-resolution OBF STEM, the H-H and T-T domain wall widths are estimated to be about 8.2 and 3.8 nm, respectively. By tDPC STEM, the positive and negative bound charge widths of the H-H and T-T domain walls are estimated to be 3.1 and 2.2 nm, respectively. Both observations consistently show the width of the H-H domain wall to be wider than that of the T-T domain wall, but the absolute values are always narrower in tDPC STEM observations. This is because the two observations are measuring different quantities. The atomic resolution OBF STEM measures the width of local polarization changes. The tDPC STEM measures the total charge density variation across the walls, that is to say the net charge resulting from the spatially overlapping polarization bound charges and screening charges, which consequently appears narrower than the expected width of the polarization bound charges alone.

The polarization bound charge densities can be estimated using formula 2 and the domain wall polarization profiles measured by atomic-resolution OBF STEM (Fig. 2, D and H) after converting the atomic column shifts to polarization units assuming a linear relation and that the magnitude of the asymptotic value inside each domain is equal to the spontaneous polarization value of bulk LiTaO_3_ at room temperature (*29*). The estimated profiles of the polarization bound charges alone are shown as blue dashed lines in Fig. 5 (B and D). To extract the spatial distribution of screening charges, the above estimated polarization bound charges are subtracted from the total charge density profiles. The results are shown in Fig. 5 (E and F). The spatial distribution of screening charges is estimated to be wider in the H-H domain wall (10.3 nm) than that in the T-T domain wall (7.7 nm). Because the magnitude of the charge per defect is four times greater for ${\text{Ta}}_{\text{Li}}^{4+}$ than it is for $V_{\text{Li}}^{-}$ , the difference in screening charge width may be related to the types of charged point defects responsible for the screening. For quantitative discussion, comparison with more elaborate theory models such as phase field simulation (*25*, *30*) may be needed but is beyond the scope of the present study.

In previous reports, interface charge accumulation is shown to be measurable by using STEM electron energy-loss spectroscopy (EELS) (*31*–*33*). To obtain enough signal, EELS measurements use relatively high electron dose conditions, typically on the order of 10^6^ electrons (e^−^)/Å^2^. In the present study, however, LiTaO_3_ is a beam-sensitive material due to the presence of Li ions. Therefore, we need to reduce the electron dose to avoid severe beam damage. In our tDPC STEM imaging, we used the electron dose condition of 4 × 10^3^ e^−^/Å^2^, which is much smaller than the typical EELS measurements. Therefore, tDPC STEM could be an alternative technique for measuring interface charge accumulation at very high spatial resolution, especially for beam sensitive materials.

In summary, the polarization-induced charges across nanoscale domain walls in ferroelectric LiTaO_3_ can be observed in real space by tDPC STEM. The present results open the possibility of directly characterizing ferroelectric interfaces and recently found unique polar textures, not only by their atomic-scale structures but also by the local charge states responsible for their unique functional properties.

## MATERIALS AND METHODS

### Sample preparation

Commercially available near-stoichiometric LiTaO_3_ single-crystal substrates were used as a starting material (Oxide Corporation, Japan). TEM specimens were prepared by mechanical polishing followed by a standard Ar-ion beam milling procedure.

### STEM observations

Atomic-resolution OBF STEM images were acquired using an aberration-corrected STEM (JEM ARM-300F, JEOL Ltd.) equipped with a second-generation segmented annular all-field (SAAF) detector (16-segmented type) (*34*). The accelerating voltage and convergence semi-angle were set to 300 kV and 30 mrad, respectively. We developed an in-house program for the real-time OBF display function and implemented it in the SAAF system (*24*). We used this real-time OBF display system to acquire all the experimental OBF images shown in the present study. The OBF images were acquired at a dwell time of 5 μs/pixel , and the total dose was set to be about 25,000 e^−^ / Å^2^.

tDPC STEM observation was conducted using the MARS microscope with a 40-segmented detector (*35*) and tilt-scan system (*17*) [JEM ARM200CF equipped with a magnetic-field-free objective lens (*36*), JEOL. Ltd.]. The 61-beam tilt conditions were generated by the tilt coils, and the tilted BF disks converged into one BF disk on the 40-segmented detector by the detilt coils. The accelerating voltage and convergence semi-angle were set to 200 kV and 1 mrad, respectively. The expected probe size is ~1 nm, and the probe current is 4.9 pA. The maximum tilt angle was about 7 mrad. The tilting step was about 1.4 mrad, and acquisition time of each tilt was 1.5 μs/pixel. For the H-H–type domain wall observation in Fig. 3, the sample thickness was estimated to be about 60 nm by EELS. For the T-T domain wall observation in Fig. 3, the sample thickness was estimated to be about 20 nm by EELS.

For the tDPC experiment, we acquired the electric field vector maps (Figs. 3, A and B, and 4, A and B) by defining the *x* axis perpendicular and the *y* axis parallel to the polarization direction. However, the observed domain walls were tilted at an angle θ with respect to the polarization direction. To correctly extract the electric field components perpendicular and parallel to the domain walls, we rotated the original (𝑥, 𝑦) coordinate system by θ to obtain a new (𝑥′, *y*′) coordinate system. The rotation is given by

$$\left(\begin{array}{c} x\prime \\ y\prime \end{array}\right)=\left(\begin{array}{cc} \text{cos}\theta & -\text{sin}\theta \\ \text{sin}\theta & \text{cos}\theta \end{array}\right)\left(\begin{array}{c} x\\ y \end{array}\right)$$ (3)

Here, the 𝑥′ axis corresponds to the electric field component perpendicular to the domain wall and the 𝑦′ axis corresponds to the electric field component parallel to the domain wall. The resulting 𝑥′ axis maps are shown in Figs. 3 (C and D) and 4 (C and D).

### Domain wall width estimation by atomic-resolution OBF STEM images

The position of each atomic column was identified by fitting a two-dimensional Gaussian function to the atomic-resolution OBF STEM images. On the basis of the identified atomic column positions, the magnitude and direction of the Ta atoms’ shifts relative to the O atomic planes were estimated. The line profiles of shift values averaged in the direction of the domain walls were fitted using the following sigmoid function

$$y=\frac{a}{1+\text{exp}\left[-b\left(x-c\right)\right]}+d$$ (4)

Using the optimized parameters, the first derivative of the sigmoid function, shown below, was used to estimate the polarization bound charge distribution profile following formula 2 and from that the domain wall width. Here, A is a normalization parameter assuming a saturation polarization of $60\;\mathrm{\mu C}/{\text{cm}}^{2}\;$ (*29*) at room temperature

$$y=\frac{A\text{exp}\left[-b\left(x-c\right)\right]}{{\left\{1+\text{exp}\left[-b\left(x-c\right)\right]\right\}}^{2}}\;$$ (5)

The domain wall width estimate was taken to be full width at tenth maximum (FWTM).

### Fitting procedures of the experimental tDPC profiles

The electric field profiles of the domain walls were formed by averaging tDPC image intensities along the direction parallel to the domain walls. The resulting electric field line profiles were fitted by the Gaussian first derivative function

$$E=-a\left(x-b\right)\text{exp}\left[\frac{-{\left(x-b\right)}^{2}}{2c^{2}}\right]$$ (6)

To account for probe blurring effects ( 2p=1.0nm ) and the blurring due to probe tilt ( 2t=thickness×sinθ ), for simplicity, we approximate these effects to be Gaussian blurring with SDs of p and t , respectively. The fitted profiles are then deconvolved with a Gaussian with SD $\sqrt{p^{2}+t^{2}}$ . Differentiating the resulting profiles, the total charge distributions of the domain walls were obtained.

By subtracting the polarization bound charges from the total charge distribution, the compensating charge distributions were estimated. The widths of the polarization-bound charges, core charges in the total charge density profiles, and compensating charge distributions were estimated by FWTM. The optimization was performed via a nonlinear least square method with a Python script using Numpy, Scipy, and PyTorch modules.

### CBED simulations

For the simulation of CBED patterns, we used software based on the Bloch-wave dynamical theory of electron diffraction [many-beam dynamical calculations and least-squares fitting (MBFIT); (*37*)].

## Supplementary Materials

### This PDF file includes:

Supplementary Note S1 Figs. S1 to S9 References
