# Supplementary material for: Real-space observation of polarization induced charges at nanoscale ferroelectric interfaces
Source: Sci Adv. 2025 Jun 13;11(24):eadu8021. doi: 10.1126/sciadv.adu8021 (PMC12164969; doi:10.1126/sciadv.adu8021)
Supplement: Supplementary file 1 — Supplementary Note S1 Figs. S1 to S9 References [file sciadv.adu8021_sm.pdf]

Science Advances
AAAS

Supplementary Materials for

Real-space observation of polarization induced charges at nanoscale ferroelectric interfaces

Masaya Takamoto *et al.*

Corresponding author: Naoya Shibata, shibata@sigma.t.u-tokyo.ac.jp; Takehito Seki, seki@sigma.t.u-tokyo.ac.jp

Sci. Adv. **11**, eadu8021 (2025)
DOI: 10.1126/sciadv.adu8021

This PDF file includes:

Supplementary Note S1
Figs. S1 to S9
References

Supplementary Note 1

Selection of optical conditions

In the present study, we selected 1 mrad convergence semiangle for the tDPC imaging. The simulated diffraction patterns for 20 nm and 60 nm thick samples using 1 mrad convergence seminangle are shown in Fig S6. In this condition, the BF and diffracted disks do not overlap, facilitating the measurement of CoM of the BF disk. Under the limited electron dose conditions, DPC/CoM imaging exhibits trade-off between the spatial resolution and the sensitivity to electric fields (38). Thus, the spatial resolution of the present optical condition is worse than our previous study (19). However, since $LiTaO_3$ is a beam sensitive material due to the presence of Li ions, we use the 1 mrad convergence semiangle to increase the sensitivity to the electric fields under the limited electron dose condition. As detailed in Fig. S4, we ultimately deconvolve the effect of probe blurring from the electric field profiles to assure fair comparison between the H-H and T-T profiles which were obtained from sample regions of different thickness.

For the tilt-scan averaging condition, we estimate the effectiveness of 61 beam tilt averaging for diffraction contrast suppression using LACBED simulation (37). Detailed analysis methods have been reported elsewhere (18). Fig. S7 shows the simulated LACBED patterns observed along the <11-20> direction. Sample thicknesses are (A) 20 nm and (B) 60 nm. The dotted red circles correspond to the CL apertures for the 61 beam tilt scan condition. It is seen that the LACBED patterns dramatically change with sample thickness. Using these LACBED patterns, we estimate the effectiveness of different beam tilt averaging conditions for diffraction contrast suppression. Here, we compare the 61 beam tilt, 25 beam tilt and 20 beam tilt precession averaging conditions shown in Fig. S8. Fig. S9 shows plots of the CoM against sample mistilt along the [1-100] direction, indicating how much diffraction contrast will arise due to sample mistilt from the zone axis orientation. For the 20 nm sample thickness, 20 beam tilt precession averaging is the most effective for suppressing the CoM increase (i.e. diffraction contrast) caused by the sample mistilt. The second most effective is the 61 beam tilt averaging and the 25 beam tilt averaging is the least effective. On the other hand, for the 60 nm sample thickness the 61 beam tilt averaging is the most effective at suppressing the diffraction contrast, whereas the 20 beam tilt precession averaging is the least effective, increasing the CoM dramatically. It should be noted that the presence and position of domain walls inside the $LiTaO_3$ crystal could not be controlled in the present study. Rather, we searched around to find H-H and T-T domain walls within the ion-thinned $LiTaO_3$ sample. Therefore, we could not control the sample thicknesses for the H-H and T-T domain wall observations. In such cases, a tilt-scan averaging condition that is robust for a wide range of sample thicknesses is desirable. The 61 beam tilt averaging is such a strategy for the present study.

In general, suitable optical conditions for tDPC experiments should be selected based on the material and quantity of interest. Theoretical estimation using LACBED can be used to estimate the robustness of the tilt-scan averaging conditions.

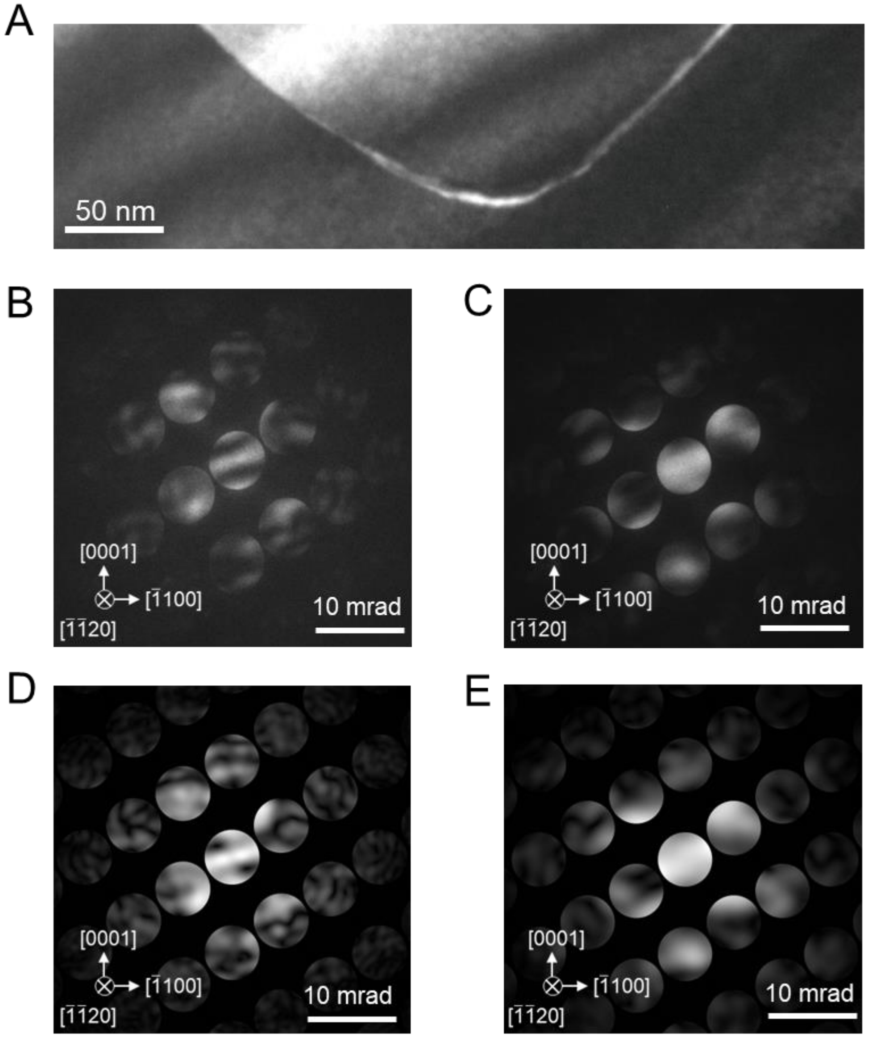

Fig. S1

CBED analysis of the $LiTaO_3$ domains in Fig.3. (**A**) Low-magnification DF TEM image of the domains and domain wall observed in Fig. 3. (**B**) Experimental CBED pattern obtained from the upper domain. (**C**) Experimental CBED pattern obtained from the lower domain. (**D**)(**E**) Theoretically simulated CBED patterns assuming the polarization directions are pointing downward and upward, respectively. Comparing the experimental and simulated CBED patterns, the domain wall is determined to be of H-H type.

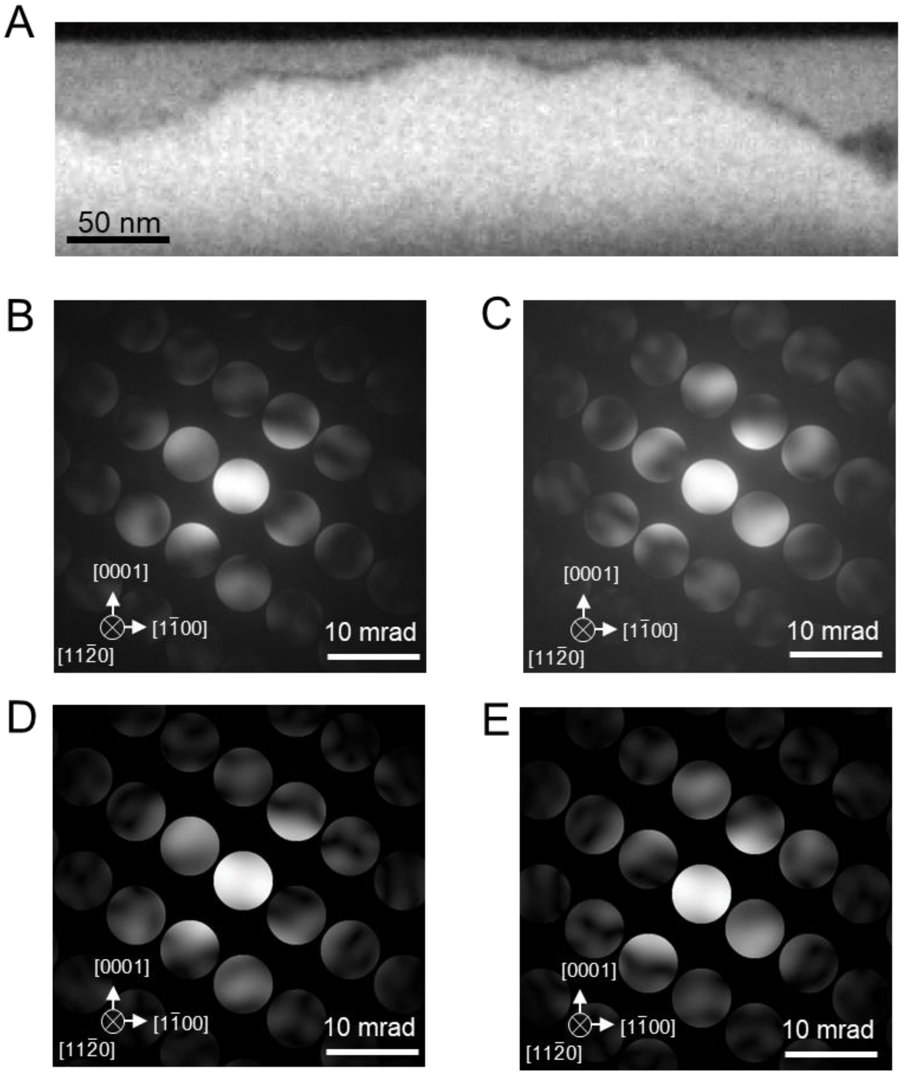

Fig. S2

CBED analysis of the $LiTaO_3$ domains in Fig.4. (**A**) Low-magnification DF TEM image of the domains and domain wall observed in Fig. 4. (**B**) Experimental CBED pattern obtained from the upper domain. (**C**) Experimental CBED pattern obtained from the lower domain. (**D**)(**E**) Theoretically simulated CBED patterns assuming the polarization directions are pointing upward and downward, respectively. Comparing the experimental and simulated CBED patterns, the domain wall is determined to be of T-T type.

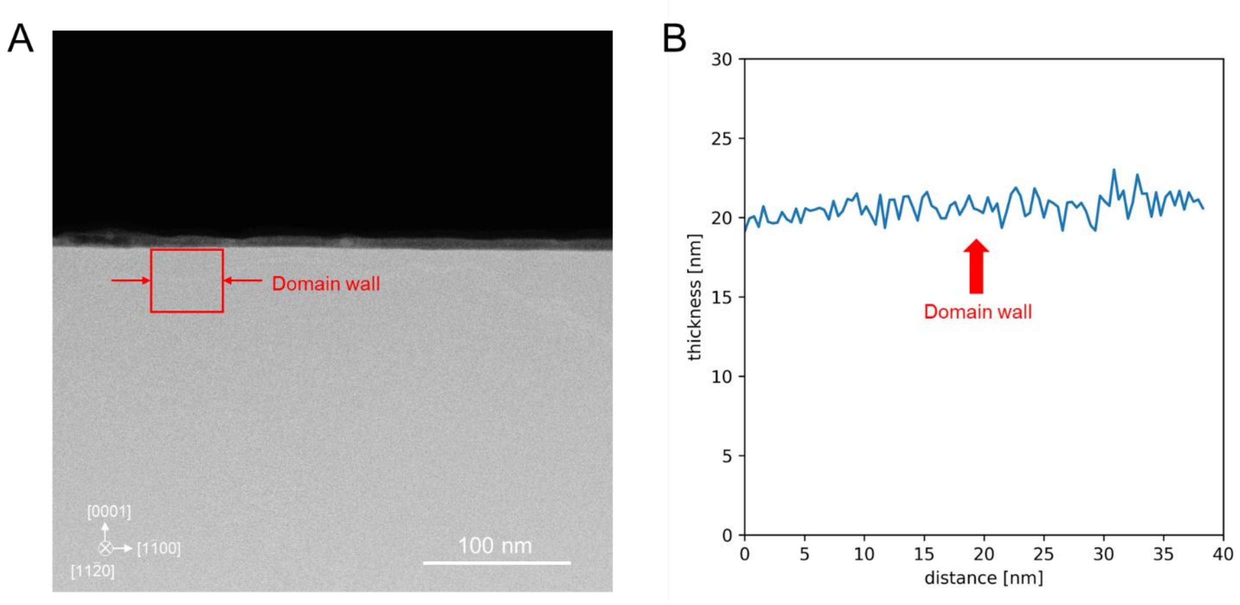

Fig. S3

Thickness across the T-T domain wall. (A) Low magnification ADF STEM image of the T-T domain wall. There is no contrast decrease around the domain wall region, indicating the absence of thickness and/or density decrease around the domain wall. A slight contrast increase can be seen, which may be due to the segregation of antisite Ta atoms and/or strain around the domain wall core region. (**B**) Sample thickness profile obtained by STEM EELS. The thickness was estimated by standard log-ratio method. It is seen that there is no sample thickness change across the domain wall.

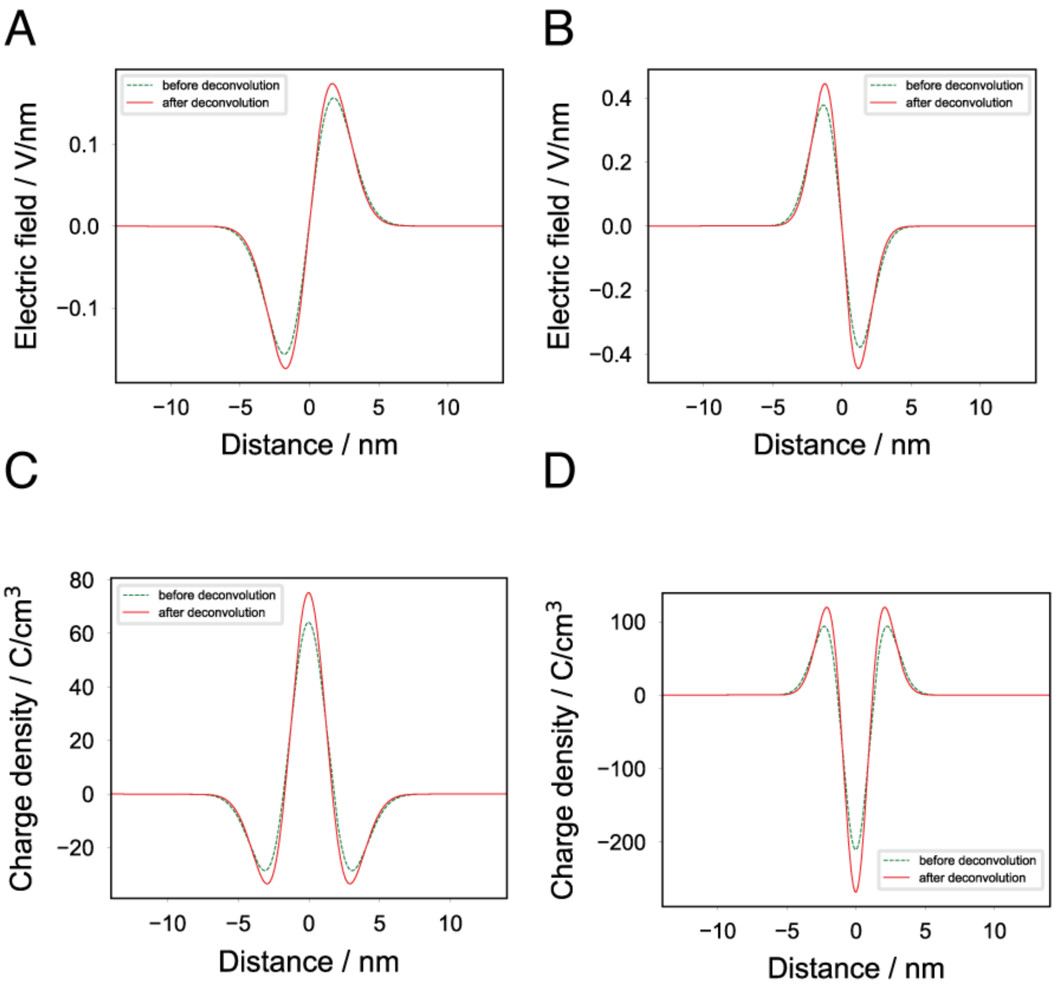

Fig. S4
Deconvolution of finite probe size and tilt blurring effect. (**A**) H-H and (**B**) T-T electric field profiles before and after deconvolving finite probe size and tilt blurring effects. (**C**) H-H and (**D**) T-T charge density profiles before and after deconvolving finite probe size and tilt blurring effect. Green dashed and red solid line in (**A**-**D**) show the profile of before and after the deconvolution processes, respectively.

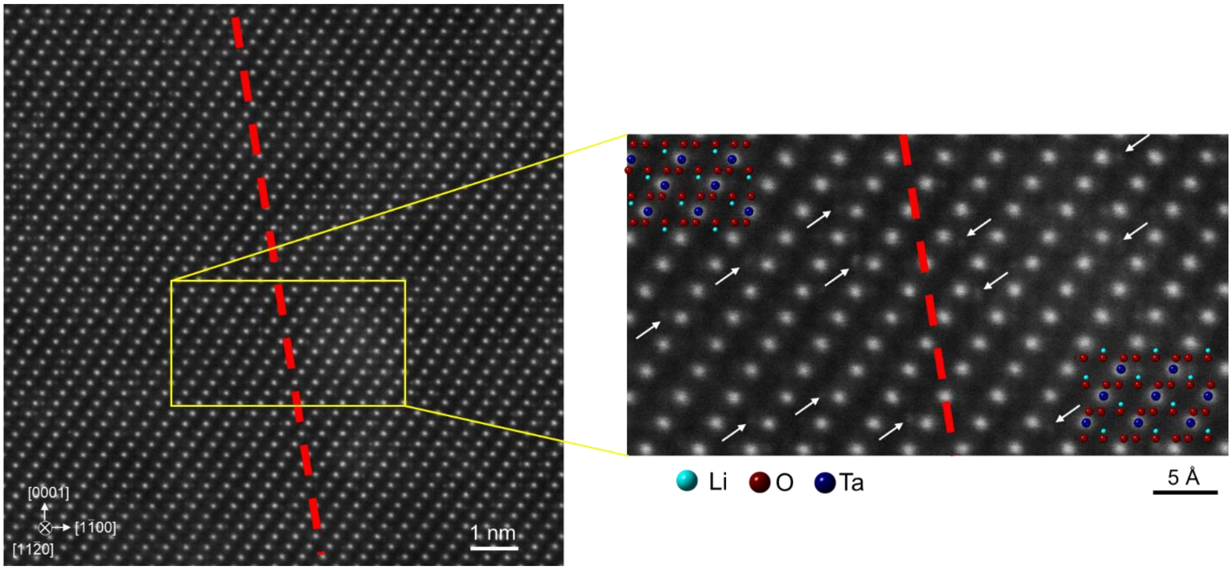

Fig. S5

ADF STEM image of the T-T domain wall core region. As indicated by the arrows, there are several Li sites which show high contrast, indicating the presence of antisite Ta^{5+} at those Li^{+} sites.

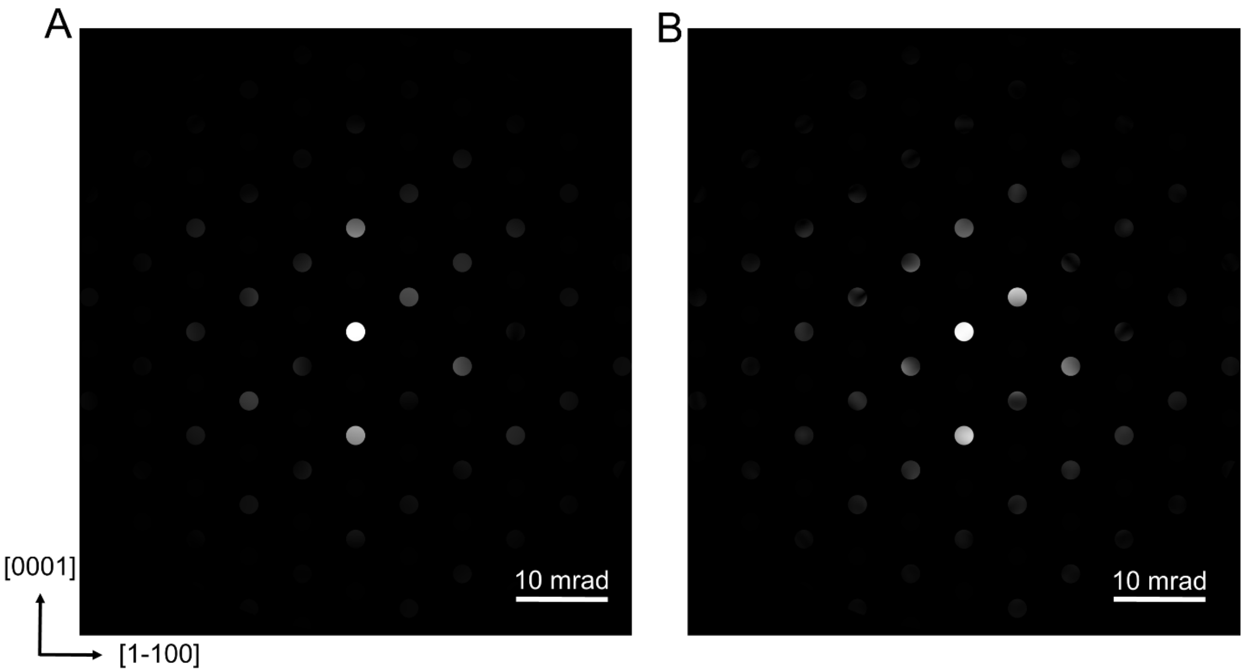

Fig. S6

Simulated CBED patterns of $LiTaO_3$ from <11-20> direction using 1 mrad convergence semiangle electron probe. (**A**) is the 20 nm sample thickness case and (**B**) is the 60 nm sample thickness case, respectively.

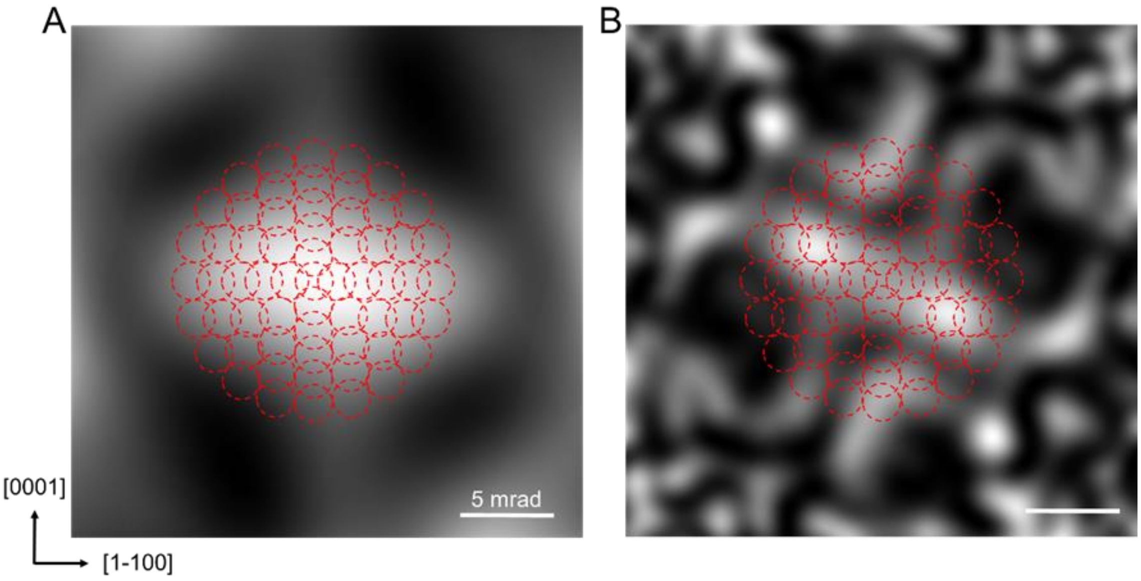

Fig. S7
Simulated LACBED patterns observed from <11-20> direction. Sample thicknesses are (**A**) 20 nm and (**B**) 60 nm, respectively. The dotted red circles are 61 tilt patterns.

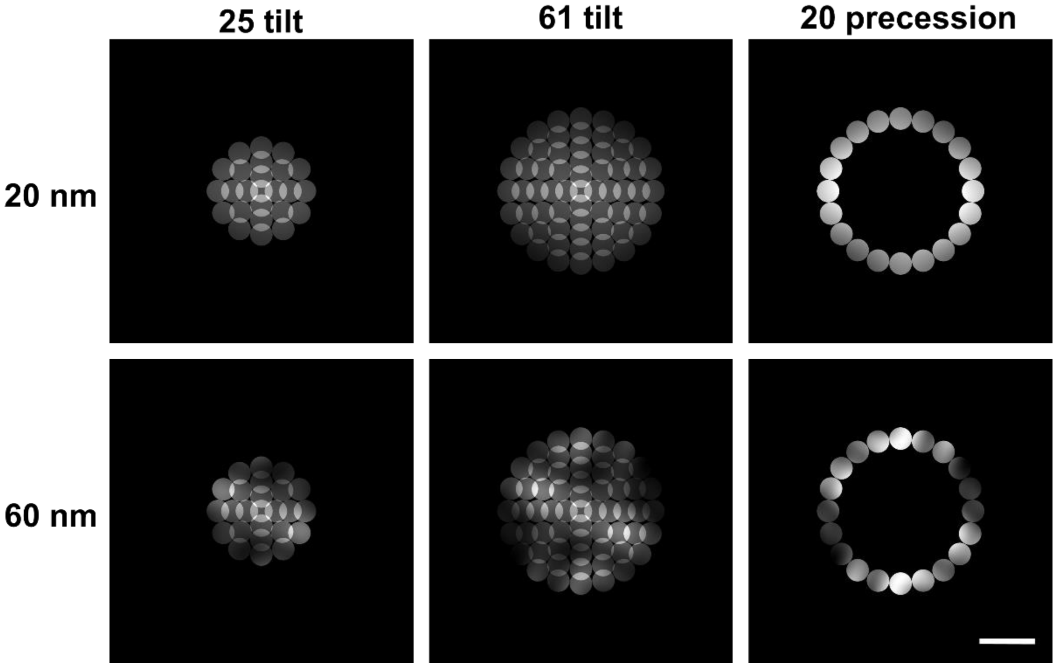

Fig. S8

Simulated 25, 61 and 20 beam presession tilt-scan patterns for 20 nm and 60 nm sample thicknesses. The individual disks shown have varying intensity as they are extracted from the corresponding tilt location in Fig. S2, their intensity variation reflecting the varying dynamical diffraction conditions with tilt. The average BF disk is formed after first aligning these patterns (achieved in experiment via the detilt coils).

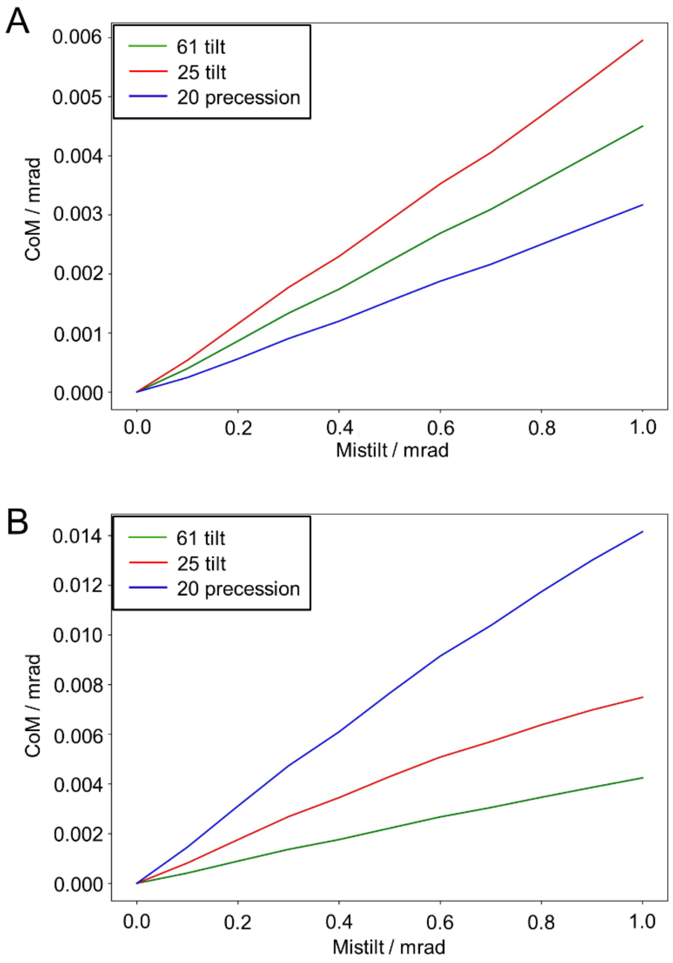

Fig. S9

Plots of the CoM against the sample mistilt along [1-101] direction. (A) is the 20 nm sample thickness case and (B) is the 60 nm sample thickness case, respectively.

REFERENCES AND NOTES

1. M. E. Lines, A. M. Glass, in *Principles, Applications of Ferroelectrics and Related Materials* (Oxford Univ. Press, 2004), pp. 82–126.

2. G. Catalan, J. Seidel, R. Ramesh, J. F. Scott, Domain wall nanoelectronics. *Rev. Mod. Phys.* **84**, 119–156 (2012).

3. C.-L. Jia, V. Nagarajan, J.-Q. He, L. Houben, T. Zhao, R. Ramesh, K. Urban, R. Waser, Unit-cell scale mapping of ferroelectricity and tetragonality in epitaxial ultrathin ferroelectric films. *Nat. Mater.* **6**, 64–69 (2007).

4. C.-L. Jia, S.-B. Mi, K. Urban, I. Vrejoiu, M. Alexe, D. Hesse, Atomic-scale study of electric dipoles near charged and uncharged domain walls in ferroelectric films. *Nat. Mater.* **7**, 57–61 (2008).

5. C.-L. Jia, K. W. Urban, M. Alexe, D. Hesse, I. Verjoiu, Direct observation of continuous electric dipole rotation in flux-closure domains in ferroelectric $Pb(Zr,Ti)O_3$. *Science* **331**, 1420–1423 (2011).

6. T. Rojac, A. Bencan, G. Drazic, N. Sakamoto, H. Ursic, B. Jancar, G. Tavcar, M. Makarovic, J. Walker, B. Malic, D. Damjanovic, Domain-wall conduction in ferroelectric $BiFeO_3$ controlled by accumulation of charged defects. *Nat. Mater.* **16**, 322–327 (2017).

7. D. Lee, H. Lu, Y. Gu, S.-Y. Choi, S.-D. Li, S. Ryu, T. R. Paudel, K. Song, E. Mikheev, S. Lee, S. Stemmer, D. A. Tenne, S. H. Oh, E. Y. Tsymbal, X. Wu, L.-Q. Chen, A. Gruverman, C. B. Eom, Emergence of room-temperature ferroelectricity at reduced dimensions. *Science* **349**, 1314–1317 (2015).

8. C. T. Nelson, B. Winchester, Y. Zhang, S.-J. Kim, A. Melville, C. Adamo, C. M. Folkman, S. H. Baek, C. B. Eom, D. G. Schlom, L.-Q. Chen, X. Q. Pan, Spontaneous vortex nanodomain arrays at ferroelectric hetero-interfaces. *Nano Lett.* **11**, 828–834 (2011).

9. Y. L. Tang, Y. L. Zhu, X. L. Ma, A. Y. Borisevich, A. N. Morozovska, E. A. Eliseev, W. Y. Wang, Y. J. Wang, Y. B. Xu, Z. D. Zhang, S. J. Pennycook, Observation of a periodic array of flux-closure quadrants in strained ferroelectric $PbTiO_3$ films. *Science* **348**, 547–551 (2015).

10. A. K. Yadav, C. T. Nelson, S. L. Hsu, Z. Hong, J. D. Clarkson, C. M. Schlepütz, A. R. Damodaran, P. Shafer, E. Arenholz, L. R. Dedon, D. Chen, A. Vishwanath, A. M. Minor, L. Q. Chen, J. F. Scott, L. W. Martin, R. Ramesh, Observation of polar vortices in oxide superlattices. *Nature* **530**, 198–201 (2016).

11. S. Das, Y. L. Tang, Z. Hong, M. A. P. Gonçalves, M. R. McCarter, C. Klewe, K. X. Nguyen, F. Gómez-Ortiz, P. Shafer, E. Arenholz, V. A. Stoica, S.-L. Hsu, B. Wang, C. Ophus, J. F. Liu, C. T. Nelson, S. Saremi, B. Prasad, A. B. Mei, D. G. Schlom, J. Íñiguez, P. García-Fernández, D. A. Muller, L. Q. Chen, J. Junquera, L. W. Martin, R. Ramesh, Observation of room-temperature polar skyrmions. *Nature* **568**, 368–372 (2019).

12. P. S. Bednyakov, B. I. Sturman, T. Sluka, A. K. Tagantsev, P. V. Yudin, Physics and applications of charged domain walls. *NPJ Comput. Mater.* **4**, 65 (2018).

13. D. Meier, S. M. Selbach, Ferroelectric domain walls for nanotechnology. *Nat. Rev. Mater.* **7**, 157–173 (2022).

14. N. Shibata, S. D. Findlay, Y. Kohno, H. Sawada, Y. Kondo, Y. Ikuhara, Differential phase-contrast microscopy at atomic resolution. *Nat. Phys.* **8**, 611–615 (2012).

15. K. Song, S. Ryu, H. Lee, T. R. Paudel, C. T. Koch, B. Park, J. K. Lee, S.-Y. Choi, Y.-M. Kim, J. C. Kim, H. Y. Jeong, M. S. Rzchowski, E. Y. Tsymbal, C.-B. Eom, S. H. Oh, Direct imaging of the electron liquid at oxide interfaces. *Nat. Nanotechnol.* **13**, 198–203 (2018).

16. F. Fujimoto, Dynamical theory of electron diffraction in Laue-case, I. General theory. *J. Phys. Soc. Japan* **14**, 1558–1568 (1959).

17. Y. Kohno, A. Nakamura, S. Morishita, N. Shibata, Development of tilt-scan system for differential phase contrast scanning transmission electron microscopy. *Microscopy* **71**, 111–116 (2022).

18. S. Toyama, T. Seki, Y. Kanitani, Y. Kudo, S. Tomiya, Y. Ikuhara, N. Shibata, Quantitative electric field mapping in semiconductor heterostructures via tilt-scan averaged DPC STEM. *Ultramicroscopy* **238**, 113538 (2022).

19. S. Toyama, T. Seki, Y. Kanitani, Y. Kudo, S. Tomiya, Y. Ikuhara, N. Shibata, Real-space observation of a two-dimensional electron gas at semiconductor heterointerfaces. *Nat. Nanotechnol.* **18**, 521–528 (2023).

20. S. Toyama, T. Seki, B. Feng, Y. Ikuhara, N. Shibata, Direct observation of space-charge-induced electric fields at oxide grain boundaries. *Nat. Commun.* **15**, 8704 (2024).

21. M. Takamoto, T. Seki, Y. Ikuhara, N. Shibata, Diffraction contrast of ferroelectric domains in DPC STEM images. *Microscopy* **73**, 422–429 (2024).

22. V. Y. Shur, Domain engineering in lithium niobate and lithium tantalite: Domain wall motion. *Ferroelectrics* **340**, 3–16 (2006).

23. K. Ooe, T. Seki, Y. Ikuhara, N. Shibata, Ultra-high contrast STEM imaging for segmented/pixelated detectors by maximizing the signal-to-noise ratio. *Ultramicroscopy* **220**, 113133 (2021).

24. K. Ooe, T. Seki, K. Yoshida, Y. Kohno, Y. Ikuhara, N. Shibata, Direct imaging of local atomic structures in zeolite using optimum bright-field scanning transmission electron microscopy. *Sci. Adv.* **9**, eadf6865 (2023).

25. M. Y. Gureev, A. K. Tagantsev, N. Setter, Head-to-head and tail-to-tail 180° domain walls in an isolated ferroelectric. *Phys. Rev. B*. **83**, 184104 (2011).

26. I. Maclaren, L. Q. Wang, D. McGrouther, A. J. Craven, S. McVitie, R. Schierholz, A. Kovács, J. Barthel, R. E. Dunin-Borkowski, On the origin of differential phase contrast at a locally charged and globally charge-compensated domain boundary in a polar-ordered material. *Ultramicroscopy* **154**, 57–63 (2015).

27. S. Kim, V. Gopalan, K. Kitamura, Y. Furukawa, Domain reversal and nonstoichiometry in lithium tantalate. *J. Appl. Phys.* **90**, 2949–2963 (2001).

28. V. Gopalan, V. Dierolf, D. A. Scrymgeour, Defect–Domain wall interactions in trigonal ferroelectrics. *Annu. Rev. Mater. Res.* **37**, 449–489 (2007).

29. T. Weigel, C. Ludt, T. Leisegang, E. Mehner, S. Jachalke, H. Stöcker, T. Doert, D. C. Meyer, M. Zschornak, Spontaneous polarization and pyroelectric coefficient of lithium niobate and lithium tantalate determined from crystal structure data. *Phys. Rev. B.* **108**, 054105 (2023).

30. E. A. Eliseev, A. N. Morozovska, G. S. Svechnikov, V. Gopalan, V. Y. Shur, Static conductivity of charged domain walls in uniaxial ferroelectric semiconductors. *Phys. Rev. B.* **83**, 235313 (2011).

31. W. Zhao, M. Li, C.-Z. Chang, J. Jiang, L. Wu, C. Liu, J. S. Moodera, Y. Zhu, M. H. W. Chan, Direct imaging of electron transfer and its influence on superconducting pairing at FeSe/$SrTiO_3$ interface. *Sci. Adv.* **4**, eeaao2682 (2018).

32. Y. Zhang, H. Lu, L. Xie, X. Yan, T. R. Paudel, J. Kim, X. Cheng, H. Wang, C. Heikes, L. Li, M. Xu, D. G. Schlom, L.-Q. Chen, R. Wu, E. Y. Tsymbal, A. Gruverman, X. Pan, Anisotropic polarization-induced conductance at a ferroelectric–insulator interface. *Nat. Nanotechnol.* **13**, 1132–1136 (2018).

33. H. Lee, N. Campbell, J. Lee, T. J. Asel, T. R. Paudel, H. Zhou, J. W. Lee, B. Noesges, J. Seo, B. Park, L. J. Brillson, S. H. Oh, E. Y. Tsymbal, M. S. Rzchowski, C. B. Eom, Direct observation of a two-dimensional hole gas at oxide interfaces. *Nat. Mater.* **17**, 231–236 (2018).

34. N. Shibata, Y. Kohno, S. D. Findlay, H. Sawada, Y. Kondo, Y. Ikuhara, New area detector for atomic-resolution scanning transmission electron microscopy. *J. Electron Microsc. (Tokyo)* **59**, 473–479 (2010).

35. Y. Kohno, T. Seki, S. D. Findlay, Y. Ikuhara, N. Shibata, Real-space visualization of intrinsic magnetic field of an antiferromagnet. *Nature* **602**, 234–239 (2022).

36. N. Shibata, Y. Kohno, A. Nakamura, S. Morishita, T. Seki, A. Kumamoto, H. Sawada, T. Matsumoto, S. D. Findlay, Y. Ikuhara, Atomic resolution electron microscopy in a magnetic field free environment. *Nat. Commun.* **10**, 2308 (2019).

37. K. Tsuda, M. Tanaka, Refinement of crystal structural parameters using two-dimensional energy-filtered CBED patterns. *Acta Crystallogr. A* **55**, 939–954 (1999).

38. S. Pöllath, F. Schwarzhuber, J. Zweck, The differential phase contrast uncertainty relation: Connection between electron dose and field resolution. *Ultramicroscopy* **228**, 113342 (2021).
